# Supplementary material for: The interactome of intact mitochondria by cross-linking mass spectrometry provides evidence for coexisting respiratory supercomplexes
Source: Mol Cell Proteomics. 2017 Dec 8;17(2):216–32. doi: 10.1074/mcp.RA117.000470 (PMC5795388; doi:10.1074/mcp.RA117.000470)
Supplement: Supplemental Data [file supp_RA117.000470_133922_0_supp_23978_fzffwf.zip › spectra_annotation/mito_spectra_annotation/402-1-4-1-2-1.pdf]

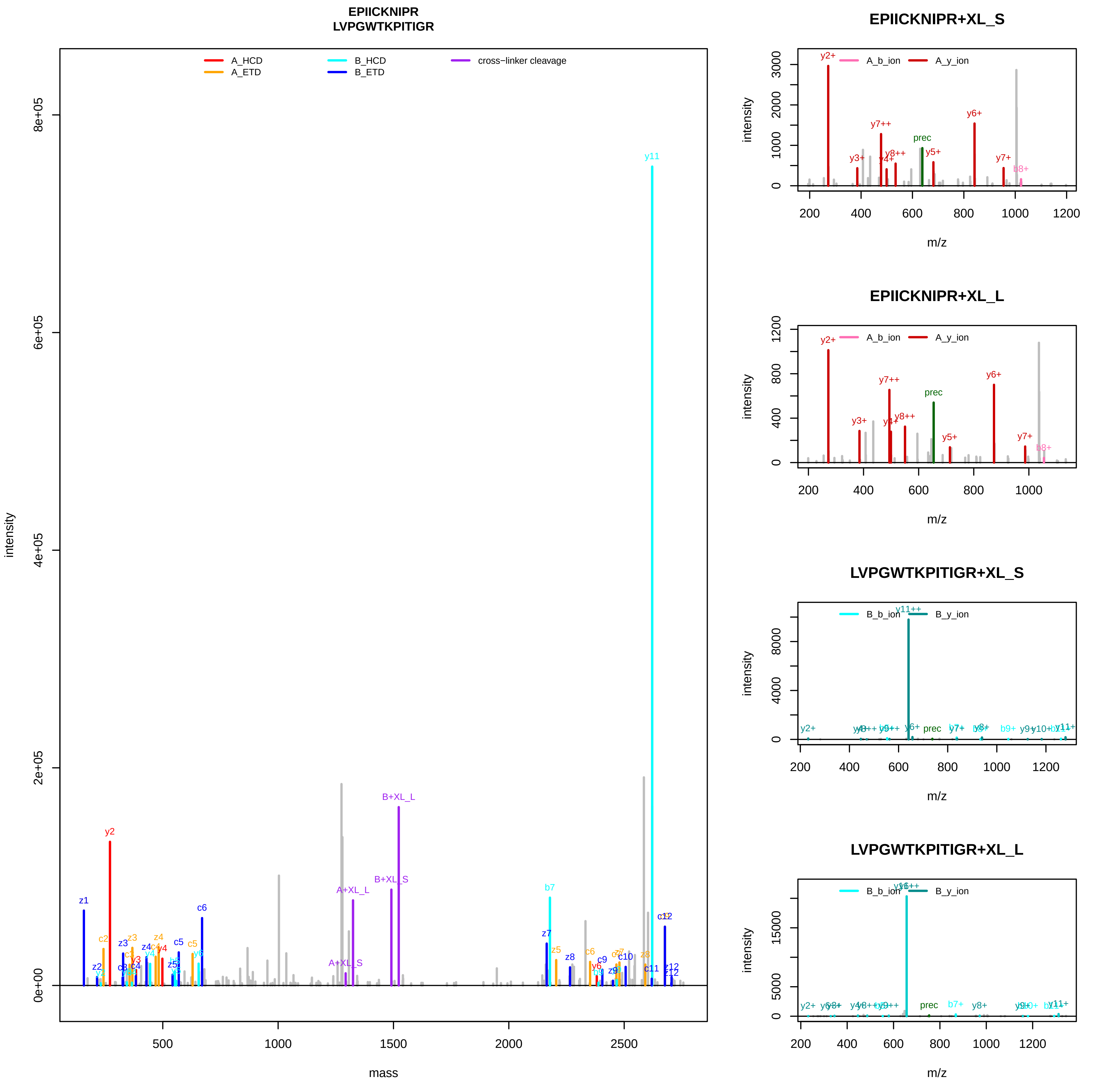

EPIICKNIPR
LVPGWTKPITIGR
A_HCD
A_ETD
B_HCD
B_ETD
cross−linker cleavage
intensity
mass
0e+00
2e+05
4e+05
6e+05
8e+05
500
1000
1500
2000
2500
y11
y2
z1
c6
c5
z3
z2
c2
z3
z4
y4
B+XL_L
B+XL_S
A+XL_L
A+XL_S
b7
z7
z8
c9
c10
c11
c12
z12
EPIICKNIPR+XL_S
A_b_ion
A_y_ion
y2+
y3+
y4+
y5+
y6+
y7+
y7++
y8++
b8+
prec
m/z
EPIICKNIPR+XL_L
LVPGWTKPITIGR+XL_S
B_b_ion
B_y_ion
y11++
prec
LVPGWTKPITIGR+XL_L
y16++
